# Supplementary material for: Indistinguishable photons from an artificial atom in silicon photonics
Source: Nat Commun. 2024 Aug 13;15:6920. doi: 10.1038/s41467-024-51265-1 (PMC11319600; doi:10.1038/s41467-024-51265-1)
Supplement: Supplementary file 1 — Supplementary Information [file 41467_2024_51265_MOESM1_ESM.pdf]

Indistinguishable photons from an artificial atom in silicon photonics: Supplementary Information

Lukasz Komza[1,2*], Polnop Samutpraphoot[2,3*], Mutasem Odeh[2,3],
Yu-Lung Tang[1,2], Milena Mathew[2,3], Jiu Chang[3], Hanbin Song[4],
Myung-Ki Kim[3,5], Yihuang Xiong[6], Geoffroy Hautier[6], Alp Sipahigil[1,2,3,†]
[1]Department of Physics, University of California, Berkeley, Berkeley, California 94720, USA
[2]Materials Sciences Division, Lawrence Berkeley National Laboratory,
Berkeley, California 94720, USA
[3]Department of Electrical Engineering and Computer Sciences,
University of California, Berkeley, Berkeley, California 94720, USA
[4]Department of Materials Science and Engineering,
University of California, Berkeley, Berkeley, California 94720, USA
[5] KU-KIST Graduate School of Converging Science and Technology,
Korea University, Seoul, 02841, Republic of Korea
[6] Thayer School of Engineering, Dartmouth College,
14 Engineering Dr, Hanover, NH 03755, USA
*These authors contributed equally to this work
†To whom correspondence should be addressed; E-mail: alp@berkeley.edu.

1 Experimental setup

The sample is mounted in a cryostat (Montana Instruments Cryostation s200) and cooled down to 3.4 K. A lensed fiber (OZ Optics TSMJ-X-1550-9/125-0.25-7-2.5-14-2) used for photon collection through the waveguide is mounted on a 3-axis nanopositioner (Attocube ANPx101/LT and ANPz102/LT) used for fiber alignment. Photons are detected by a pair of SNSPDs (Quantum Opus QO-NPD-1200-1600), each with 60% detection efficiency at optimal polarization.

We calibrate our fiber coupling efficiency by injecting laser light into port P2 (Fig. S1) and measuring the reflected power after the first beamsplitter. We calculate the waveguide-fiber coupling efficiency η_{fc} from

$$P_{\mathrm{out}} = \left(\eta_{\mathrm{fc}}^2 RT\right) P_{\mathrm{in}} \tag{1}$$

where R and T are the measured reflection and transmission coefficiencts of the beamsplitter. We measure $\eta_{\mathrm{fc}} \approx 0.5$ for TE-polarized light. The photonic crystal end mirror is reflective for TE polarization, and maximizing the reflected power allows us to selectively excite TE-polarized light. The excitation laser beam is sent through the top vacuum window of the cryostat and focused by a microscope objective (Mitutoyo LCD Plan Apo NIR 50, NA=0.42) mounted on a 3-axis translation stage (Sutter Instrument MP-285) used for raster scanning. Measurements in Fig. 1(c), Fig. 2(b,c), and Fig. 3(b,d,e) are performed with continuous wave 635 nm excitation (Thorlabs S1FC635), whereas measurements in Fig. 2(d), Fig. 3(c), and Fig. 4(b) are performed with 705-715 nm pulsed Ti:sapphire laser (Coherent Chameleon Ultra II). The pulsed laser has a pulse duration of $140\,\mathrm{fs}$ and repetition rate of 80 MHz. To perform experiments at $40\,\mathrm{MHz}$, the laser was downsampled using an electro-optic modulator to suppress every other pulse. We observed finite pulse suppression, with 8.0 dB extinction of suppressed pulses, resulting in weak but observable contributions in our measurements at odd multiples of the repetition period (Fig. 3(c), Fig. 4(b)). We tuned the photon indistinguishability in the HOM experiment by aligning the relative polarization of the two arms of the MZI interferometer to be parallel and perpendicular. To achieve this, we artificially broadened a tunable O-band laser (Santec TSL-570) to eliminate interference effects while aligning the polarization. When we inject a broadband light source into the MZI interferometer in Fig. 4, if the two arms have orthogonal polarizations, the output orthogonal polarization is completely unpolarized. If the polarizations are parallel the output state is completely polarized. Therefore, we measure the degree of polarization (DOP) and tune it to be either 0% or 100% using a fiber polarization

controller in one arm of the interferometer. We use a polarimeter (ThorLabs PAX1000IR2) to measure the DOP of MZI output with broadband light. We achieved DOPs within 5% of 0% and 100%, where the DOP drifted slowly due to polarization drifts from thermal fluctuations.

2 G center yield in devices

We implant carbon at a fluence of $10^{12}\,\mathrm{cm}^{-2}$, and observe one G center in a $100\,\mu\mathrm{m}$-long waveguide. This allowed us to investigate a single spatially isolated G center, but alternate recipes may produce dramatically higher yields. We find that carbon implantation at $10^{13}\,\mathrm{cm}^{-2}$ and rapid thermal annealing at 1000°C for 20 seconds followed by 200 keV proton irradiation at $10^{13}\,\mathrm{cm}^{-2}$ produces more G centers in waveguides than we can spatially (0.42 NA) or spectrally (0.1 nm) resolve in our experimental setup. We performed additional experiments that corroborate past reports of a threshold temperature above which G centers are annealed out[1], with no measurable bulk G center signal following a rapid thermal annealing at 350°C for 20 seconds. We note that for the device used in the manuscript our PECVD oxide was deposited at 350°C over 3 hours, which we expect to have further reduced the yield. We do not expect this threshold temperature to impact compatability with silicon photonics fabrication, as G center formation can be performed as a final step in device fabrication.

3 Carbon diffusion in silicon

We used Stopping and Range of Ions in Matter (SRIM) simulations to estimate the depth distribution of carbon atoms in the silicon device layer after ion implantation at 36 keV and 7° tilt. The SRIM simulations gave a mean depth of 112 nm and a longitudinal straggle of 41 nm. We used finite difference method to estimate the carbon distribution after annealing. We used a diffusion coefficient of carbon in silicon $D = 0.33\ e^{-2.92eV/kT}\mathrm{cm}^2/\mathrm{s}$ from Ref. [2]. Our simulation results in Fig. S4 show that after our $1000\,^{\circ}\mathrm{C}$ thermal anneal for 20 s, the carbons

are uniformly distributed inside the 220 nm device layer. These results indicate that choosing an annealing temperature of $900\,^{\circ}\mathrm{C}$ should maintain emitter localization near the center of the waveguide where the mode intensity is maximum. Further SIMS measurements are needed to develop an improved understanding of carbon diffusion and G center formation. These simulations suggest that the G center in our experiment could be positioned at any depth inside the waveguide.

4 First-principles modeling of G centers

We performed first-principles calculations using Vienna *ab-initio* simulation package (VASP)[3,4] with the projector augmented-wave method (PAW)[5]. All calculations were spin-polarized with a plane wave cutoff energy of 400 eV. The Heyd-Scuseria-Ernzerhof (HSE)[6] functional with 25% exact exchange was used to provide an improved description of the electronic structures to the semilocal functionals. The G center was positioned in a 512-atom supercell and a Γ-only k-point sampling. The supercell was optimized at a fixed volume until the forces on the ions were smaller than 0.01 eV/Å. The single-particle Kohn-Sham levels of the G center at its $1A'$ ground state are shown in Fig. S5. We used the so-called configuration B of the G center as multiple reports indicate that it is the configuration in best agreement with experimental data[7–9]. We found that in its ground state, the defect with symmetry C_{1h} introduces two highly localized defect levels where the a'' lying below the valence band maximum and the a' within the band gap, 1 eV above the valence band. This single-particle picture agrees with other hybrid computations when taking into account slight differences in methodology and supercell size[7,8,10]. It disagrees quantitatively with G_0W_0 which places the unoccupied state significantly lower in energy[11]. We tentatively attribute this disagreement to the sensitivity of G_0W_0 to its starting wavefunctions obtained in the generalized gradient approximation (GGA). We use constrained-HSE to simulate the $1A''$ excited state of the G center as shown in Fig. S5. We

have performed the excitation by emptying the localized a' defect state below the valence band and occupying the a'' state. We noted that the resulting single-particle hole state a'' in the excited state moved slightly above the valence band edge, similar to negatively charged splitting vacancy in the diamond[12,13]. The ZPL can be obtained from the energy difference between the total energy of the excited and the ground states. This methodology has been shown to give ZPLs within 100 meV from experiment for defects in diamond[13,14]. Our computed ZPL energy of the intra-defect transition is 1000 meV, which is in reasonable agreement with the experimental measurement of 968 meV in Fig. 2. The ground state single-particle diagram (Fig. S5) suggests that an alternative excitation mechanism to an intra-defect transition would be to excite an electron from the valence band to the localized a'' defect state forming a bound exciton defect. Bound exciton defects have been suggested in the T center in silicon[15,16]. The computed ZPL for these valence band excitations are from 937 to 958 meV. These excitations show a much lower calculated transition dipole moment (0.65 to 0.83 D) than the intra-defect transition (2.4 to 3.3 D) due to the very different nature of the states (delocalized to localized). Accordingly, the computed radiative lifetime of the intra-defect transition is 0.15 to 0.3 μs, which is an order of magnitude smaller than that of the valence band excitations (3.59 to 3.69 μs). The intra-defect transition results in a much smaller radiative time in better agreement with the clear and bright PL of the G center. Our analysis suggests therefore that localized defect states from a' to a'' are responsible for the PL of the G center. We note that emission from defect bound excitonic-like recombination to valence band states could be present in the PL spectra but not observable due to the phonon sideband and their much weaker signal. Additionally, our computations indicate that the transition dipole moment is aligned along the $\langle 110 \rangle$ direction.

5 Efficiency analysis

We define the system efficiency $\eta = 0.4(1) \times 10^{-3}$ as the probability of detecting a ZPL photon per excitation:

$$\eta = \eta_{\text{QE}} \eta_{\text{wg}} \eta_{\text{filter}}^{\text{BP}} \eta_{\text{network}} \tag{2}$$

where η_{QE} is the quantum efficiency, η_{wg} is the probability of an excited G center emitting a photon into the waveguide mode, $\eta_{\text{filter}}^{\text{BP}}$ is the efficiency of our spectral filtering setup, and η_{network} is the efficiency of our fiber network and detectors. We did not use the Fabry-Perot cavity during calibration measurements to reduce calibration uncertainties originating from the emitter linewidth. We assume that the emitter is excited with unit efficiency with each laser pulse. We estimate the quantum efficiency using

$$\eta_{\text{QE}} = \frac{\eta}{\eta_{\text{wg}} \eta_{\text{filter}}^{\text{BP}} \eta_{\text{network}}} \tag{3}$$

We simulate $\eta_{\text{wg}} \leq 0.8$, where the inequality is due to the uncertainty of the position of the emitter position. The filtering efficiency $\eta_{\text{filtering}} \approx 0.14$ is a product of the ZPL branching ratio (0.18) and bandpass filter transmission (0.8). We measure the efficiency of the remaining components in our setup to be $\eta_{\text{network}} \approx 0.2$, originating from a combination of losses in lensed fiber coupling efficiency (0.5), finite detector efficiency (0.6), and the remaining optical components in the fiber network. From these calibrations, we can bound the quantum efficiency of the G center as $\eta_{\text{QE}} > 0.02$, where the inequality is due to the uncertainty in the emitter position. We can put a corresponding upper bound on the radiative lifetime ($\tau_r = \gamma_r^{-1} < 260$ ns) using:

$$\eta_{\text{QE}} = \frac{\gamma_r}{\gamma_{nr} + \gamma_r} \tag{4}$$

where γ_r and γ_{nr} are the radiative and nonradiative decay rates, $\Gamma_G = \gamma_r + \gamma_{nr} = (4.6\text{ns})^{-1}$. We note that previous experiments[17] found a tighter upper bound of $\tau_r < 74$ ns. The estimated lifetime from our first principles calculations is between 150 and 300 ns.

6 Time-resolved two-photon interference

Our experimental two-photon interference results in Fig. 4 show that the successive photons emitted from a single G center in a photonic waveguide show a high degree of indistinguishability. We describe a model that explains the observed temporal dynamics which indicate that spectral fluctuations in G centers shows long-time correlations. We follow the time-resolved description of two-photon quantum interference in Ref.[18], and adapt it for exponential wavepackets. We consider the time-resolved dynamics of two single-photon pulses simultaneously arriving at the beamsplitter (at $z = 0$). These two pulses are created with a 25 ns delay at the source G center, and simultaneously arrive at the beamsplitter due to the delayed interforemeter setup shown in Fig. 4. The two spatio-temporal mode function amplitudes for the photons at the position of the beamsplitter ($z = 0$) are:

$$\psi_{1,2}(t, z = 0) = \frac{1}{\sqrt{T_1}} \exp(-t/(2T_1) - i\omega_{1,2}t) \tag{5}$$

where ω_1 and ω_2 are the carrier frequencies of the first and second single-photon pulses respectively, and T_1 is the emission lifetime as measured in Fig. 2. The emission lifetime T_1 is a result of homogeneous broadening based on radiative γ_r and non-radiative decay γ_{nr} and is therefore constant between successive pulses. We consider the case where the two photons can be at different frequencies. Here, we make a quasistatic approximation where each photon pulse has a well defined frequency (ω_i) and frequency fluctuations might occur between different pulses. The origin of frequency differences between successive pulses is discussed below. The joint photon-detection probability due to the interference of two single-photon wavepackets is given by:

$$P_{\text{joint}}(t_0, t_0 + \tau) = \frac{1}{4} |\psi_1(t_0 + \tau)\psi_2(t_0) - \psi_2(t_0 + \tau)\psi_1(t_0)|^2 \tag{6}$$

For the exponential single-photon wavepackets in our experiment (Eq. 5, Fig. 2), the joint photon detection probability becomes

$$P_{\text{joint}}(t_0, t_0+\tau) = \frac{e^{(-2t_0-\tau)/T_1}}{2T_1^2}(1-\cos\Delta\tau) \tag{7}$$

and simplifies to

$$P_{\text{joint}}(\tau) = \frac{e^{-\tau/T_1}}{4T_1}(1-\cos\Delta\tau) \tag{8}$$

if the detuning Δ between the two photons is constant.

Photons can be made distinguishable at very large detunings ($\Delta \to \infty$) or by having orthogonal polarizations. In Eq. 8, the distinguishable case corresponds to setting the cosine term equal to zero. We therefore define a parameter χ to describe the degree of indistinguishability of the single photons generated.

$$P_{\text{joint}}(\tau) = \frac{e^{-\tau/T_1}}{4T_1}(1-\chi\cos\Delta\tau) = \alpha g^{(2)}(\tau) \tag{9}$$

where $\chi_{\text{ideal}} = 1$ for indistinguishable and $\chi_{\text{ideal}} = 0$ for distinguishable photons. We experimentally tune the indistinguishability by adjusting the relative polarization of the incoming photons to be parallel or orthogonal. This allows us to use experimentally measured values to quantify the degree of indistinguishability:

$$\chi_{\text{exp}} = 1 - g^{(2)}_{\parallel}(0)/g^{(2)}_{\perp}(0) \tag{10}$$

In the curve in Fig. 4, we see an exponential feature around $g^{(2)}_{\parallel}(0)$ instead of a cosine as suggested by Eq. 9. The experimentally observed exponential behavior near $|\tau| < 1$ ns is caused by fluctuations in the detuning (Δ_{12}) between successive pulses (ψ_1, ψ_2). For solid-state quantum emitters, the dominant source of spectral broadening ($\Gamma = \gamma_r/2 + \gamma_{nr}/2 + \gamma_d$) beyond the lifetime limit ($\gamma_r + \gamma_{nr}$) is caused by spectral diffusion. Spectral diffusion is a pure dephasing process (γ_d) where emission frequency fluctuates due to external classical noise

sources such as fluctuating charges in the solid-state environment. Such charge fluctuations cause a frequency shift on the optical transition frequencies via the DC Stark shift[19]. Fig. 3(a) shows that the broadening in our system is well-captured by a model where the single-photon emission frequency is sampled from a Lorentzian distribution

$$p(\omega_i) = \frac{\Gamma}{2\pi} \frac{1}{(\omega_i - \omega_0)^2 + (\Gamma/2)^2} \tag{11}$$

with a full width half maximum of $\Gamma/2\pi = 2.8$ GHz. If the emission frequency of two successive pulses are uncorrelated, the relative detuning Δ_{12} between successive pulses will be sampled from a Lorentzian distribution at twice the single-photon linewidth $\Gamma^{\text{uncorr}} = 2\,\Gamma$. If the correlation timescale (τ_c) of the emitted photon frequencies is longer than the two-photon delay ($\delta\tau = 25$ ns in the experiment), we expect

$$p(\Delta_{12}) = \frac{\Gamma^{\text{HOM}}}{\pi} \frac{1}{\Delta_{12}^2 + (\Gamma^{\text{HOM}})^2} \tag{12}$$

where the effective two-photon linewidth in the experiment is $\Gamma^{\text{HOM}} < 2\,\Gamma$. In our experiment, $\Gamma \approx \gamma_d$ since γ_d is much greater than $\gamma_r + \gamma_{nr}$, indicating that the effective optical linewidth at the experimental time scale is $(\tau_{\text{HOM}})^{-1}/2\pi$. Finally, we can obtain the experimentally observed $g^{(2)}(\tau)$ temporal dynamics by using the quasistatic approximation, and integrating Eq. 9 with the probability distribution for two photon detunings in Eq. 12

$$G^{(2)}(\tau) = \int_{-\infty}^{\infty} d\Delta_{12} P_{\text{joint}}(\Delta_{12}, \tau) p(\Delta_{12}) \tag{13}$$

$$= \frac{e^{-\tau/T_1}}{4T_1} \left(1 - \chi e^{-(\Gamma^{\text{HOM}}\tau)}\right) \tag{14}$$

We fit the data to the functional form of $G^{(2)}(\tau)$ + b.g. where b.g. is a variable to account for background noise. We use the value of $T_1 = 4.6(1)$ ns from the lifetime measurements in Fig. 3. We find $(\Gamma^{\text{HOM}})^{-1}$ =0.4(1) ns. The effective two-photon linewidth in the quantum interference measurement is $\Gamma^{\text{HOM}}/2\pi = 0.4(1)$ GHz, about an order of magnitude smaller than the measured linewidth of the emission spectrum in Fig. 3. We attribute this to the time dynamics of

spectral diffusion in the system, as Fig. 3(b) was acquired over a ten-minute period, while we interfere two subsequently emitted photons separated by 25 ns in our HOM experiment.

Based on Eq. 10, any detuning (or linewidth) between the two photon pulses should still lead to high indistinguishability at $\tau = 0$. However, this requires the inverse linewidth and the interference window to be longer than the detector and electronics timing jitters. We measured the timing jitter of our SNSPDs by measuring laser-laser correlations and extracting σ from a gaussian fit. We obtained a jitter of 252 ps. We include this in our model by convolving Eq. 14 with a gaussian curve with σ = 252 ps. In the main text, we report $g^{(2)}(0)$ and HOM visibility values based on the raw data, shot noise estimates, and Eq. 10, without any dependence on model details or timing jitter correction. We estimate the contribution to a non-zero HOM dip from the timing jitter of our SNSPDs to be 48%. Other factors limiting the depth of our HOM dip include dark counts, tails from adjacent peaks due to our finite lifetime-to-repetition period ratio, and imperfect polarization overlap, for which we estimate contributions of 39%, 8%, and 5% respectively.

References

1. Davies, G. The optical properties of luminescence centres in silicon. *Phys. Rep.* **176,** 83–188 (1989).
2. Newman, R. & Wakefield, J. The diffusivity of carbon in silicon. *J. Phys. Chem. Solids* **19,** 230–234 (1961).
3. Kresse, G. & Furthmüller, J. Efficiency of ab-initio total energy calculations for metals and semiconductors using a plane-wave basis set. *Comput. Mater. Sci.* **6,** 15–50. ISSN: 0927-0256 (1996).
4. Kresse, G. & Furthmüller, J. Efficient iterative schemes for ab initio total-energy calculations using a plane-wave basis set. *Phys. Rev. B* **54,** 11169–11186 (1996).
5. Blöchl, P. E. Projector augmented-wave method. *Phys. Rev. B* **50,** 17953–17979 (1994).
6. Heyd, J., Scuseria, G. E. & Ernzerhof, M. Hybrid functionals based on a screened Coulomb potential. *J. Chem. Phys.* **118,** 8207–8215 (2003).

7. Ivanov, V. *et al.* Effect of Localization on Photoluminescence and Zero-Field Splitting of Silicon Color Centers. Preprint at https://arxiv.org/abs/2206.04824 (2022).

8. Udvarhelyi, P., Somogyi, B., Thiering, G. ő. & Gali, A. Identification of a Telecom Wavelength Single Photon Emitter in Silicon. *Phys. Rev. Lett.* **127,** 196402 (2021).

9. Song, L. W., Zhan, X. D., Benson, B. W. & Watkins, G. D. Bistable interstitial-carbon–substitutional-carbon pair in silicon. *Phys. Rev. B* **42,** 5765–5783 (1990).

10. Wang, H., Chroneos, A., Londos, C. A., Sgourou, E. N. & Schwingenschlögl, U. G-centers in irradiated silicon revisited: A screened hybrid density functional theory approach. *J. Appl. Phys.* **115,** 183509 (2014).

11. Timerkaeva, D., Attaccalite, C., Brenet, G., Caliste, D. & Pochet, P. Structural, electronic, and optical properties of the C-C complex in bulk silicon from first principles. *J. Appl. Phys.* **123,** 161421 (2018).

12. Gali, A. & Maze, J. R. Ab initio study of the split silicon-vacancy defect in diamond: Electronic structure and related properties. *Phys. Rev. B* **88,** 235205 (2013).

13. Gali, A., Janzén, E., Deák, P., Kresse, G. & Kaxiras, E. Theory of Spin-Conserving Excitation of the $N - V^-$ Center in Diamond. *Phys. Rev. Lett.* **103,** 186404 (2009).

14. Li, S., Thiering, G., Udvarhelyi, P., Ivády, V. & Gali, A. Carbon defect qubit in two-dimensional WS2. *Nat. Commun.* **13** (2022).

15. Bergeron, L. *et al.* Silicon-Integrated Telecommunications Photon-Spin Interface. *PRX Quantum* **1,** 020301 (2020).

16. Dhaliah, D., Xiong, Y., Sipahigil, A., Griffin, S. M. & Hautier, G. First-principles study of the T center in silicon. *Phys. Rev. Materials* **6,** L053201 (2022).

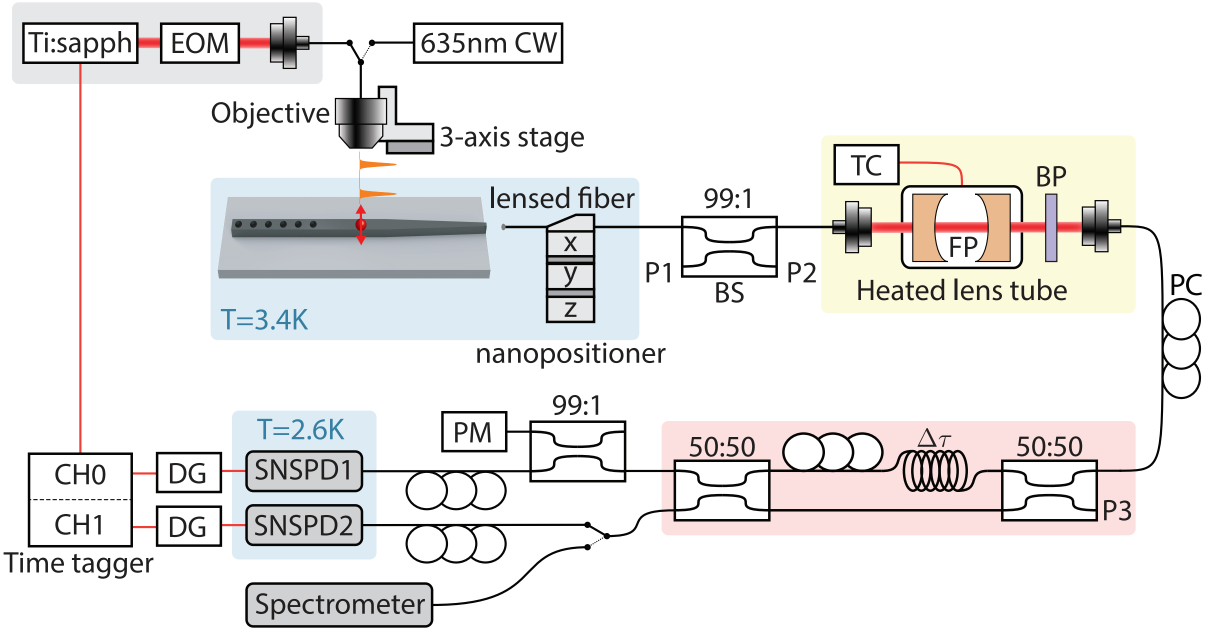

Supplementary Fig. 1: Experimental setup details. Black and orange lines represent optical fiber and electrical connections respectively. Empty ports of fiber beamsplitters (P1, P2, P3) were used for efficiency calibrations, such as measuring the fiber coupling or transmission through the fabry-perot cavity. EOM: electro-optic modulator. 635 nm CW: 635 nm continuous-wave laser. BS: beamsplitter. TC: temperature controller. FP: Fabry Perot cavity. BP: bandpass filter. PC: polarization controller. PM: polarimeter. SNSPD: superconducting nanowire single-photon detector. DG: delay generator. CH0/CH1: time tagger channels. $\Delta\tau$ = 25 ns delay line.

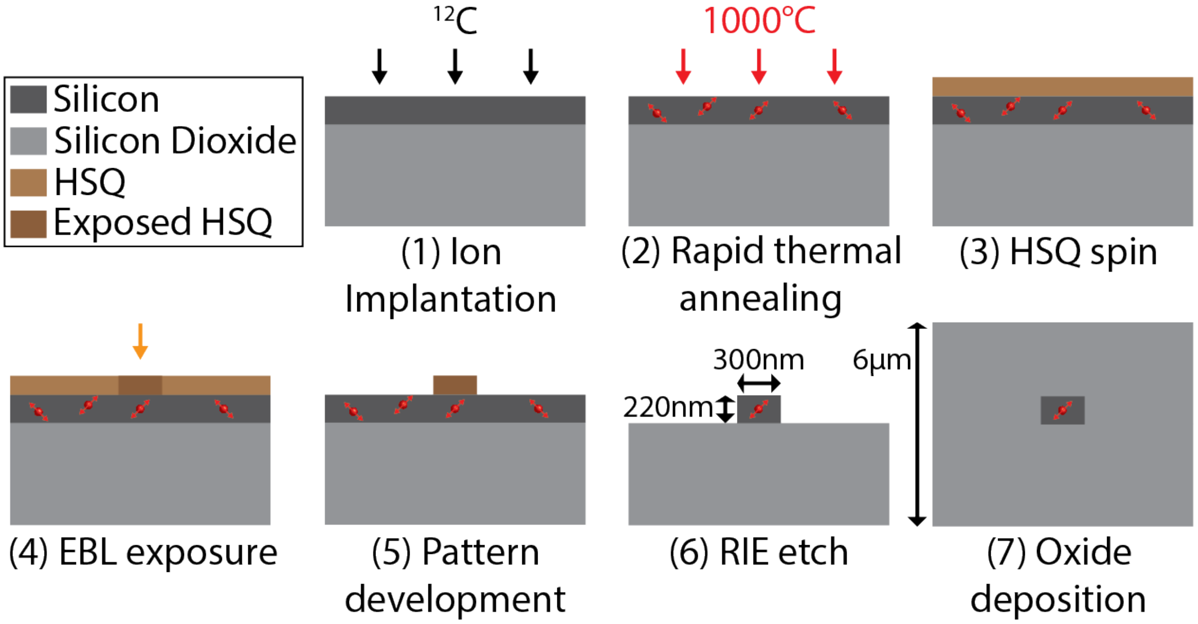

Supplementary Fig. 2: Device fabrication process. All photonics fabrication is done post-implantation. After oxide deposition, the waveguide facets are defined by cleaving the chip.

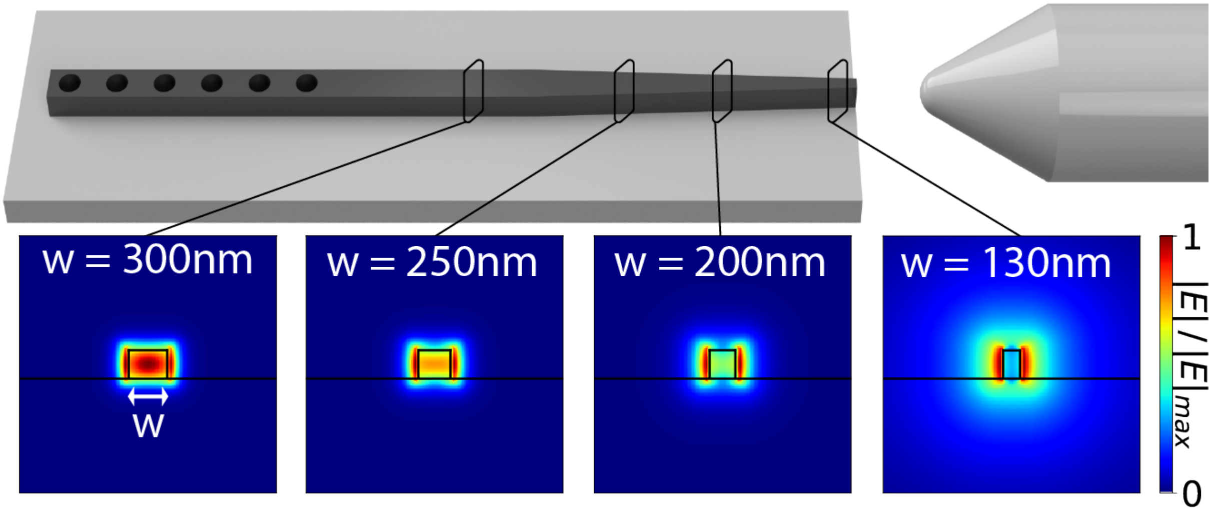

Supplementary Fig. 3: Adiabatic mode coupler. The waveguide width is tapered to mode match with the lensed fiber, which has a mode field diameter (MFD) of $2.5\,\mu\mathrm{m}$. The adiabatic tapering region is $50\,\mu\mathrm{m}$ long to have high efficiency. The emitter shown in the manuscript is in the $300\,\mathrm{nm}$ wide region to achieve stronger coupling to the waveguide.

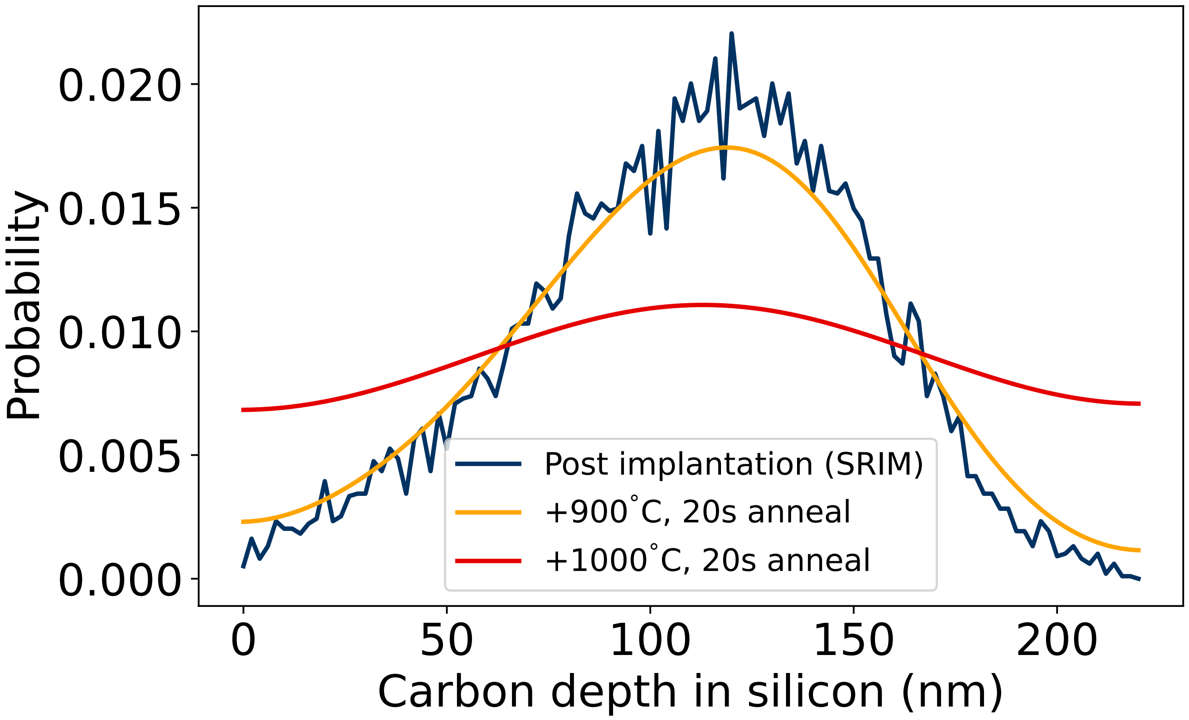

Supplementary Fig. 4: Simulation of carbon distribution in silicon after implantation and rapid thermal annealing. In our experiments, we used a $1000\,^{\circ}\mathrm{C}$ thermal anneal for 20 s. Simulations based on diffusion coefficients in the literature suggest a near-uniform depth distribution of carbon inside the silicon waveguide.

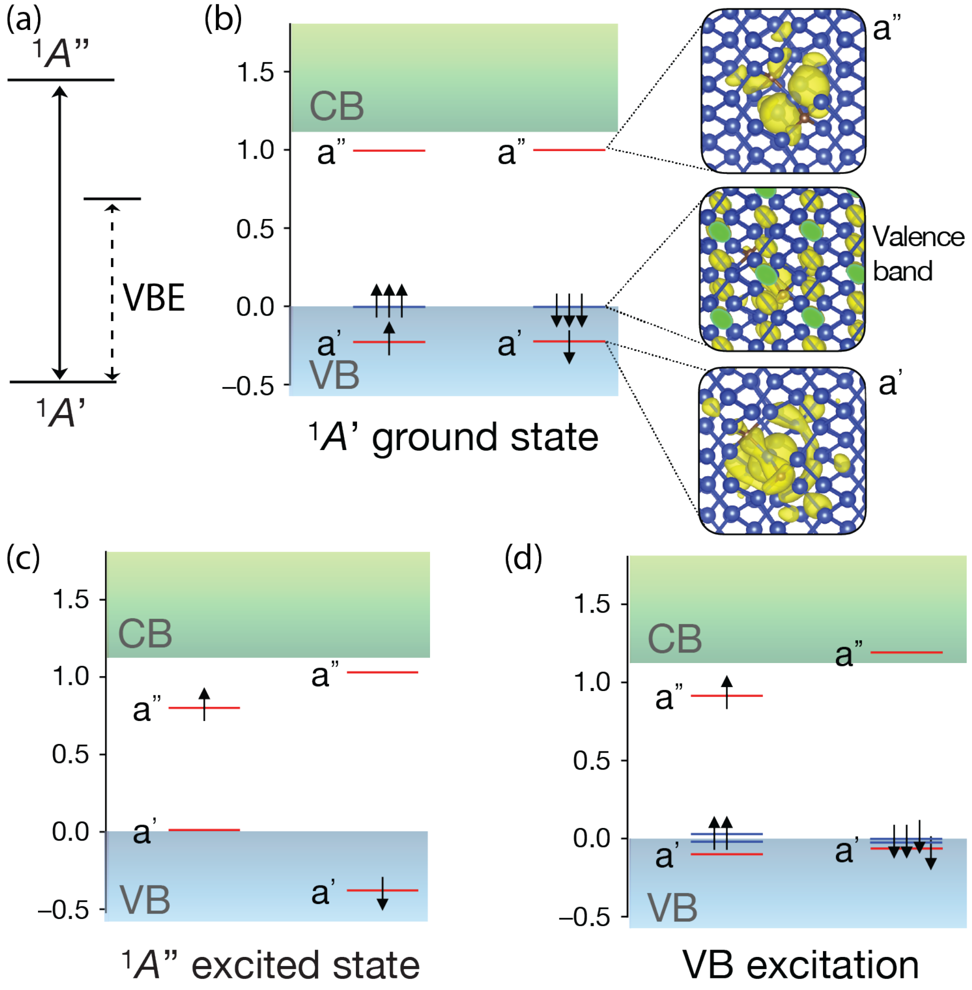

Supplementary Fig. 5: Optical transitions in G centers from first principles. (a) Radiative transitions of the G center from the $1A'$ ground state. $1A' \leftrightarrow 1A''$ are between localized defect levels. (b) The single-particle levels of the G center in its $1A'$ ground state. The localized defect states (a', a'') are shown in red, and the host valence band (VB) states are shown in blue. The localized orbitals a' and a'' are shown as insets with an isocontour value of 0.0005 a^{-3}. The host valence band orbitals show delocalized character with an isocontour value of 0.0001 a^{-3}. (c) The $1A''$ excited state is obtained by constraining the occupation of the unoccupied orbitals that creates the a' hole state and populates the a'' state in the gap. (d) Radiative transition from a valence band state (VBE). The single-particle levels of G center where the radiative recombination is through the localized in gap-defect state a'' and the delocalized host state (blue).
